# Strength-based assessment for future violence risk: a retrospective validation study of the Structured Assessment of PROtective Factors for violence risk (SAPROF) Japanese version in forensic psychiatric inpatients

**DOI:** 10.1186/s12991-018-0175-5

**Published:** 2018-01-30

**Authors:** Hiroko Kashiwagi, Akiko Kikuchi, Mayuko Koyama, Daisuke Saito, Naotsugu Hirabayashi

**Affiliations:** 1 Department of Forensic Psychiatry, National Center Hospital of Neurology and Psychiatry, 4-1-1, Ogawahigashicho, Kodaira, Tokyo 187-8553 Japan; 2 Department of Forensic Psychiatry, National Institute of Mental Health, National Center of Neurology and Psychiatry, Kodaira, Tokyo Japan

**Keywords:** Protective factor, SAPROF, Violence, Risk assessment, Forensic inpatients

## Abstract

**Background:**

The Structured Assessment of PROtective Factors for violence risk (SAPROF) was recently developed as a strength-based addition to the risk assessment of future violent behavior. We examined the interrater reliability and predictive accuracy of the SAPROF for violence in forensic mental health inpatient units in Japan.

**Methods:**

This retrospective record study provides an initial validation of the SAPROF in a Japanese sample of 95 forensic psychiatric inpatients from a complete 2008–2013 cohort. Violent outcomes were assessed 6 and 12 months after hospitalization.

**Results:**

We observed moderate-to-good interrater reliability for the SAPROF total score and the internal factors, motivational factors, external factors, and the Final Protection Judgment scores. According to a receiver operating characteristic analysis, the SAPROF total score and all subscale scores predicted violence at both 6 and 12 months after hospitalization with high accuracy. Furthermore, the predictive validity of a combination of the SAPROF with the Historical Clinical Risk Management-20 (HCR-20) outperformed that of the HCR-20 alone.

**Conclusions:**

The results provide evidence of the value of considering protective factors in the assessment of future violence risk among Japanese forensic psychiatric inpatients. The SAPROF might allow for a more balanced assessment of future violence risk in places where the population rates of violent crime are low, such as Japan, but a validation study in a different setting should confirm this. Moreover, future studies should examine the effectiveness of treatment and promoting community re-integration on motivating patients and treatment staff.

## Background

Over the last few decades, our knowledge of violence risk assessment and the risk factors for violence have increased markedly. Risk-focused assessment tools, such as the Historical Clinical Risk Management-20 (HCR-20) [1], are widely used in forensic settings worldwide. However, very little attention has been paid to the factors that might compensate for these risk factors and thereby reduce the risk of violence recidivism, namely protective factors.

According to the manual of the Structured Assessment of PROtective Factors for violence risk (SAPROF), protective factors refer to any characteristic of a person, his/her environment, or his/her situation that reduces the risk of future violent behavior [2, 3]. The identification of specific protective factors is a major challenge for the future [4–6]. A balanced risk assessment involves the evaluation of both risk and protective factors. In other words, when these protective factors are not considered, risk assessment becomes unbalanced, thereby leading to inaccurate predictions [5]. Further, this might lead to pessimism among both offenders, who are often stigmatized, and therapists, which might lead to the long-term detention of forensic psychiatric patients. Protective factors might explain the reason for the lack of recidivism in some high-risk individuals [7], such as individuals with severe psychopathy. As the reduction of violent re-offenses is a major goal of treatment, interventions should not focus on merely curtailing risk factors, but also on strengthening protective factors [8, 9]. Moreover, insight into the presence or absence of protective factors might offer a complete view of the individual in their context and provide guidelines for treatment and risk management. The standardized assessment of protective factors might also have a positive and motivating effect on both patients and treatment staff [2, 3]. Therefore, such a strength-based approach might be particularly effective when integrated into psychosocial treatment, such as that which uses a problem-solving approach and seeks empowerment (e.g., the Good Lives Model) [10, 11].

Inspired by past research and reinforced by the desire of clinicians to focus more on the changeable positive factors in risk assessment, de Vogel and colleagues [2, 3] developed the SAPROF, a positive, dynamic addition to the collection of structured risk assessment tools. The SAPROF is a checklist of 17 protective factors identified in a literature review on the protective and contextual factors of future violence [12], contextual factors related to violent recidivism, and the clinical experience of the mental health professionals and researchers at the Van der Hoeven clinic in the Netherlands [2, 3]. Two of the factors are considered static and 15 dynamic, and its overall aim is to inform clinicians of potential goals for treatment intervention. The SAPROF might offer valuable guidance in narrowing the gap between risk assessment and violence prevention [13]. The SAPROF validation study further revealed that it has a good interrater reliability and good predictive validity for forensic inpatients and outpatients [2, 3, 13].

In Japan, there are no widely used structured risk assessment tools for violence. In accordance with a new mental health act (the Medical Treatment and Supervision Act [MTSA], 2005), the Ministry of Health, Labour and Welfare developed and introduced 17 specific risk assessment items (Kyoutu Hyouka Koumoku in Japanese) for common points of view among various professionals. These items share many commonalities with the items of the HCR-20 [1]. Of course, in addition to the focus on problem extraction and evaluation from a negative perspective, there have been voices pointing out the importance of positive evaluations that focus on protective factors. Consequently, from the initial implementation of the MTSA, the necessity of attending to this positive perspective of future violence assessment, on which the SAPROF is based, and treatment through a recovery model has been recognized. Still, evaluation tools based on this perspective do not currently exist, which has necessitated the development of the SAPROF Japanese Version.

In this study, a Japanese translation of the SAPROF was completed, and its back translation was subsequently certificated by the original authors. We then sought to validate the measure in Japanese forensic settings by examining its predictive accuracy for violent incidents among forensic psychiatric inpatients in Japan.

## Methods

We conducted a retrospective record study of a complete cohort of patients admitted to the National Center Hospital of Neurology and Psychiatry, which has 66 beds dedicated to forensic psychiatric patients.

### Participants

In Japan, individuals who have committed serious harm to others while in a state of insanity or diminished responsibility because of a mental disorder receive a court order for hospitalization that is pursuant to the MTSA. Forensic psychiatric wards are dedicated to the containment and treatment of such individuals. In this study, we included all such patients admitted to the forensic psychiatric wards of the National Center Hospital of Neurology and Psychiatry between April 2008 and November 2012, and followed them up through November 2013. Patients who were hospitalized for less than 1 year were excluded because we used two observation periods for the occurrence of violence: 6 and 12 months.

### Diagnosis

Participants were diagnosed by a consulting psychiatrist according to the International Classification of Diseases, Tenth Edition (ICD-10) criteria [14]. The classification was based on single-digit ICD-10 codes (F0 to F9), and when a participant had multiple psychiatric diagnoses, we included only the primary diagnosis, consistent with the previous validation study on the HCR-20 in Japanese forensic inpatients [15]. We determined which diagnosis was considered primary based on which diagnosis was directly connected to the offense for which the patient was hospitalized.

### Assessment

#### SAPROF

The SAPROF is a checklist of protective factors that is intended for use in conjunction with structured professional judgment risk assessment tools, such as the HCR-20. The SAPROF comprises 17 protective factors (see Table 1), all of which are rated on a three-point scale (0 = the protective factor is clearly absent or there is no evidence that the protective factor is present, 1 = the protective factor may be present or is present to some extent, 2 = the protective factor is clearly present) reflecting the extent to which they are present for a given patient in a specific situation. After rating all the protective factors, a Final Protection Judgment score, which reflects the degree of protection against relapse into violence, is rated on a five-point scale (low, low–moderate, moderate, moderate–high, high). The SAPROF items are organized into three scales: internal factors, motivational factors, and external factors. Items 1 and 2 (internal factors) are considered static, whereas the other 15 factors are dynamic and, therefore, changeable during treatment. Items 3–14 are expected to improve during treatment because higher scores on these factors indicate greater balance in internal and social functioning and increased motivation. Items 15–17, by contrast, are expected to decrease during treatment because they relate to the protection offered by external professional care, which is expected to be reduced as much as possible by the end of treatment. A total SAPROF protection score is the sum of the scores of the 17 items. The total SAPROF internal, motivational, and external scores are the sums of the five (items 1–5), seven (items 6–12), and five (items 13–17) item scores in these factors, respectively. The total SAPROF protection score ranges from 0 to 34.

**Table 1:** The SAPROF-17 factors and expected changes during treatment

	Expected changes during treatment
Internal factors	
1. Intelligence	Static
2. Secure attachment in childhood	Static
3. Empathy	Improving
4. Coping	Improving
5. Self-control	Improving
Motivational factors	
6. Work	Improving
7. Leisure activities	Improving
8. Financial management	Improving
9. Motivation for treatment	Improving
10. Attitudes toward authority	Improving
11. Life goals	Improving
12. Medication	Improving
External factors	
13. Social network	Improving
14. Intimate relationship	Improving
15. Professional care	Decreasing
16. Living circumstances	Decreasing
17. External control	Decreasing

The procedure for translating the SAPROF into Japanese was as follows. First, the original English version was translated into Japanese by the first author (HK), the second author (AK), the last author (NH), and five others. Second, back translation was done by a native English speaker, whose second language was Japanese. Finally, the back-translated version of the SAPROF was confirmed and approved by the researchers who had originally developed the SAPROF in the Netherlands.

The SAPROF was completed based on a psychiatric evaluation report recorded by a psychiatrist, a life and environmental report recorded by a probation officer, and clinical records of multi-disciplinary professionals within the first 2 weeks following hospitalization. The SAPROF was scored by the first author (HK), who is a forensic psychiatrist and had attended English and Japanese training sessions for scoring the SAPROF. To establish interrater reliability, 30 randomly selected cases were coded independently by two different raters, HK and MK (the latter of whom is a clinical psychologist and was trained to score the SAPROF).

#### HCR-20

The HCR-20 is a commonly used risk-focused, structured professional judgment assessment tool for future violence. We used the HCR-20 version 2, Japanese Edition [15]. The HCR-20 comprises 20 items across the following subscales: historical (10 items), clinical (five items), and risk management (5 items). Each item is scored on a three-point scale as 0 (absent), 1 (possibly present or present only to a limited extent), or 2 (present). The risk management items are scored separately for the likelihood of institutional (In) and community (Out) violence. In the current study, we included only the scores for the risk of institutional violence because all the participants were inpatients. Psychopathy was omitted from the scale following the Japanese HCR-20 version 2 validation study [15] because there is evidence that it adds little to this assessment, and guidelines warn against the use of this item unless psychopathy is rated using the Hare Psychopathy Checklist-Revised (PCL-R) [16]. There is no validation study on the PCL-R Japanese version. Furthermore, high-psychopathic inpatients are very rare in the MTSA wards in Japan because these individuals tend to be sentenced to prison, as they are regarded to assume full responsibility for their offense. Consequently, the HCR-20 total score ranges from 0 to 38.

As described above, designated evaluation items (Kyoutu Hyouka Koumoku) are typically used in Japanese forensic units, whereas HCR-20 scores are recorded only for research and are not intended for clinical practice in Japan. The HCR-20 version 2, Japanese Edition was administered by trained psychiatrists at admission, 3, 6 months, and 1 year after hospitalization, as well as at discharge. In our study, only HCR-20 admission scores were used because we also administered the SAPROF at admission. We obtained these data for the HCR-20 from original validation study conducted by Arai et al. [15].

We also calculated an overall total risk and protection score by subtracting the SAPROF total score from the HCR-20 total score: this HCR-20–SAPROF total score ranges from − 34 to 38.

#### Violent outcomes

Data on the incidents of violence in forensic psychiatric wards were obtained from the patients’ electronic medical records. We searched these records for the following: (1) records with tags related to interpersonal violence; (2) records with tags related to loss of impulse control; (3) records associated with advanced observation level; and (4) records related to administration of restraint or seclusion. This search protocol yielded almost all incidents of violence during hospitalization. In their introduction to the HCR-20, Webster et al. [1] defined violence as actual, attempted, or threatened harm to another individual. Thus, any behavior directed at a typical person that would make that person experience fear can be regarded as violence. More specifically, we used the following definition of violence based on the validation study of the HCR-20 (version 2) Japanese Edition [15]:

1. An act attempting to harm another person physically (e.g., striking, kicking, biting, or throwing something at another person);
2. Intentional destruction of property in front of others (e.g., breaking glass or slamming a table to the floor);
3. Sexual offenses/harassment.

Although threats of harm are regarded as “verbal violence,” they are not always documented in medical records [17]. Therefore, they were excluded from the present study.

Violent incidents were measured at 6 and 12 months after hospitalization. Violent incidents that occurred within 6 months of hospitalization were included in both the 6- and 12-month analyses. The data on violence were collected independently from the HCR-20 and SAPROF data by different researchers, each blind to others’ ratings.

#### Data analysis

The interrater reliability of the SAPROF was examined via the intraclass correlation coefficient (ICC) using a two-way random-effect variance model that assesses the consistency of agreement [18]. The critical values for single-measure ICCs are as follows: ICC ≥ 0.75 = excellent; 0.60 ≤ ICC < 0.75 = good; and 0.40 ≤ ICC < 0.60 = moderate [19].

The predictive validity of the SAPROF and HCR-20 and the combined score of both measures were examined at 6 and 12 months using receiver operating characteristic (ROC) analyses. The ROC curve is created by plotting the sensitivity against the specificity at various cut-off points. The predictive ability of the measure is determined by the area under the curve (AUC), with 0.5 representing a prediction no better than chance and 1 representing a perfect positive prediction. An AUC greater than 0.71 is regarded as a large effect size [20]. Note that values on the SAPROF (total score, subscale scores, and Final Protection Judgment) do not reflect the risk of violent incidents but, rather, their absence. Conversely, values on the HCR-20 and the HCR-20–SAPROF are considered to reflect risk of violent incidents.

## Results

### Background characteristics and occurrence of violence

During the study period, 128 patients were admitted. Eight patients were excluded because of insufficient data, and 25 patients were excluded because they were discharged within 1 year. Thus, a total of 95 patients were included in analyses.

Table 2 shows the demographic characteristics of the participants. All the participants were Asians and Japanese-speaking adults (≥ 20 years old). Most of them had been diagnosed with a schizophrenic disorder, while the second most frequent diagnosis involved mental and behavioral disorders resulting from psychoactive substance use. No participant had a primary diagnosis of a personality disorder. The index offense of most participants involved interpersonal violence.

**Table 2:** Participant demographic characteristics

	*n* = 95	
Age		
	Mean age (SD)	45.73 (14.12)
	20–29	12 (12.6%)
	30–39	28 (29.4%)
	40–49	21 (22.1%)
	50–59	17 (17.9%)
	60–69	10 (10.5%)
	70–79	6 (6.3%)
	80–89	1 (1.1%)
Sex		
	Male	83 (87.4%)
	Female	12 (12.6%)
Diagnosis		
	F00-09	2 (2.1%)
	F10-19	14 (15.8%)
	F20-29	70 (73.7%)
	F30-39	6 (6.3%)
	F60-69	1 (1.1%)
	F80-89	1 (1.1%)
Index offence		
	Murder	35 (36.8%)
	Bodily injury	33 (34.7%)
	Arson	18 (18.9%)
	Sexual offence	3 (3.2%)
	Robbery	6 (6.3%)

Diagnostic categories are based on ICD-10 codes as follows: F00–09: organic, including symptomatic, mental disorders; F10–19: mental and behavioral disorders because of psychoactive substance use; F20–29: schizophrenia, schizotypal, and delusional disorders; F30–39: mood [affective] disorders; F60–69: disorders of adult personality and behavior; F80–89: disorders of psychological development.

Scores on the SAPROF, HCR-20, and HCR-20–SAPROF, as well as incidents of violence following hospitalization, are shown in Table 3. Electronic medical records showed that at 6 and 12 months, 11 (11.6%) and 17 (17.9%) of the patients, respectively, had committed at least one violent act.

**Table 3:** SAPROF, HCR-20, and HCR-20-SAPROF scores and the occurrence of violence

	6 months	12 months
Number	95	95
Interpersonal violence	9	13
Property destruction	2	4
Sexual violence	0	0

	6 months			12 months		
Violence	Yes	No	*P*	Yes	No	*P*
Number	11	84^a^		17	78^a^	
SAPROF total (SD)	12.1 (1.9)	17.4 (4.1)	< 0.001	12.7 (2.3)	17.6 (4.1)	< 0.001
Internal (SD)	2.0 (0.9)	4.0 (1.8)	< 0.001	2.1 (1.0)	4.1 (1.8)	< 0.001
Motivational (SD)	3.8 (1.8)	6.4 (2.2)	< 0.001	4.2 (1.6)	6.5 (2.2)	< 0.001
External (SD)	6.3 (0.5)	7.0 (0.9)	0.015	6.4 (0.5)	7.0 (0.9)	0.006
Final judgment (SD)	1.8 (0.8)	2.9 (0.9)	< 0.001	1.9 (0.7)	3.0 (0.9)	< 0.001
HCR-20 (SD)	24.7 (4.3)	18.6 (6.4)	0.003	22.2 (5.4)	18.7 (6.5)	0.045
HCR-20-SAPROF (SD)	12.6 (3.9)	1.3 (9.1)	< 0.001	9.5 (6.1)	1.1 (9.3)	< 0.001

*T* tests were calculated to compare group means

^a^One participant’s intelligence could not be evaluated because of the patient’s refusal to take an IQ test, and one participant did not take any medications, so the number of participants contributing to the mean SAPROF internal and motivational scores in the nonviolent group was 83 at 6 months and 77 at 12 months, while that contributing to the mean SAPROF total score in the nonviolent group was 82 at 6 months and 76 at 12 months

### Reliability

Cronbach’s alpha of the whole SAPROF was 0.81 (data available for 93 participants). The interrater reliability analyses of the randomly selected 30 cases are shown in Table 4. They revealed single-measure ICCs for the total SAPROF score, the internal score, the motivational score, the external score, the Final Protection Judgment score, and for the individual items in the SAPROF. The professional care, living circumstances, and external control items (all external factors) were scored 2 for all participants because all the participants were hospitalized, treated, and under observation according to a court order.

**Table 4:** Interrater reliability of the SAPROF

Scale	ICC	*P*
SAPROF total	0.70	< 0.001
Internal	0.78	< 0.001
Motivational	0.57	< 0.001
External	0.76	< 0.001
Final judgment	0.60	< 0.001
Items		
Intelligence	0.96	< 0.001
Secure attachment in childhood	0.71	< 0.001
Empathy	0.71	< 0.001
Coping	0.32	0.042
Self-control	0.44	0.007
Work	0.46	0.004
Leisure activity	0.32	0.041
Financial management	0.36	0.024
Motivation for treatment	0.043	0.410
Attitude toward authority	0.50	0.002
Life goals	0.53	0.001
Medication	0.51	0.002
Social network	0.72	< 0.001
Intimate relationship	0.81	< 0.001

The critical values for single-measure ICCs are as follows: ICC ≥ 0.75 = excellent; 0.60 ≤ ICC < 0.75 = good; and 0.40 ≤ ICC < 0.60 = moderate. The professional care, living circumstances, and external control items (all external factors) were excluded because they were scored 2 for all participants

### Predictive accuracy of the SAPROF for violence

Table 5 shows the results of the ROC analysis of the predictive accuracy of the SAPROF, HCR-20 and HCR-20–SAPROF at 6 and 12 months. Six months after hospitalization, the AUCs for the total SAPROF score and for the internal factors, motivational factors, external factors, Final Protection Judgment, HCR-20, and HCR-20–SAPROF scores were all > 0.71. Twelve months after admission, all scores were > 0.71, except for that of the HCR-20 (AUC = 0.67). Notably, the predictive validity of the combined HCR-20–SAPROF outperformed the predictive validity of the HCR-20 alone at both 6 and 12 months after admission.

**Table 5:** ROC analysis of the predictive accuracy of the SAPROF, HCR-20, and HCR-20–SAPROF for violence

Observation period	Scale	Area under the curve	95% confidence interval
6 months	SAPROF total	0.87	0.79–0.95
	Internal	0.83	0.73–0.93
	Motivational	0.82	0.70–0.94
	External	0.74	0.60–0.89
	Final judgment	0.82	0.69–0.95
	HCR-20	0.79	0.69–0.90
	HCR-20–SAPROF	0.87	0.79–0.94
12 months	SAPROF total	0.85	0.77–0.94
	Internal	0.83	0.74–0.92
	Motivational	0.8	0.70–0.91
	External	0.72	0.59–0.85
	Final judgment	0.82	0.72–0.92
	HCR-20	0.67	0.54–0.80
	HCR-20–SAPROF	0.78	0.67–0.88

The values on the SAPROF (total score, subscale scores, and Final Protection Judgment) do not reflect risk of violent incidents but, rather, their absence. Conversely, values on the HCR-20 and the HCR-20–SAPROF reflect risk of violent incidents

## Discussion

This is the first study to examine the predictive ability of the SAPROF for future violence (i.e., the absence of violence) in a sample of forensic psychiatric inpatients in Japan. The interrater reliability analysis indicated that there was moderate-to-good reliability for the total SAPROF score, as well as the scores on the three subscales (internal, motivational, and external factors) and the Final Protection Judgment score. Furthermore, according to the ROC analysis, the total SAPROF score, as well as the scores on the three subscales and the Final Protection Judgment score, predicted the absence of violence at 6 and 12 months with high accuracy.

The fact that the SAPROF Japanese version had predictive validity is consistent with a previous study conducted among a Dutch sample of inpatients [21]. In this Dutch inpatient study, most of the patients (89%) had been diagnosed with at least one personality disorder (particularly Cluster B disorders), while 53% of the patients had been diagnosed with a major mental illness (primarily psychotic disorders, such as schizophrenia) [21]. The duration of observation for violence was 12 months after the initial assessment. While they included verbal aggression as well as physical aggression (e.g., hitting, pushing) among the incidents of violence, the overall observed violent incident rate was 11%. In the present study, most participants (73.7%) had been diagnosed with a schizophrenic disorder, suggesting that the diagnostic characteristics are different from the Dutch sample; nevertheless, the observation duration and violent incident rate were similar to that sample. One possible reason for this might be that differences in the treatment environment and management skills affected the violence rate. Abidin et al. [22], in a prospective study conducted in Ireland, reported that the total score on the SAPROF predicted the absence of violence (AUC = 0.847) in forensic inpatients at 6 months after admission, with a violent incident rate of 13.3%. In that study, most participants (85%) had a primary diagnosis of either schizophrenia or schizoaffective disorder [22].

The predictive validity results are not, however, entirely consistent with a Swiss retrospective cross-validation study [23], which showed that the total SAPROF score had an AUC of 0.70, while the total HCR-20 score had an AUC of 0.85 for violent and sexual incidents 3 years after release. In that study, almost half the participants were sex offenders, 58.8% had been diagnosed with a personality disorder, and 27% had mental retardation, whereas only 5.9% had exhibited psychosis. In addition, about 30% of the offenders were reconvicted within 3 years of their release.

Singh et al. [24] reported that the rates of violence in persons identified as high risk by structured risk assessment instruments showed substantial variation. In addition, they suggested that the rates were elevated when the population rates of violence were higher, when a structured professional judgment instrument was used, and when there was a lower population of men in a study. Given that population rates of violent crime in Japan are low in comparison with other countries (as reported by the Ministry of Justice) [25], we can infer that using only risk-focused assessment tools might increase the rates of false positives. Thus, the HCR-20 might not be sufficient if used alone. Assessing protective factors alongside risk factors might thus help in providing more accurate and balanced predictions of future violence in Japan. Our findings encourage clinicians working in forensic psychiatric settings to take these protective factors into account when assessing violence risk. Additionally, focusing on strengths or protective factors might be useful for psychosocial treatment, particularly by motivating both clinicians and patients. Future studies on the effectiveness of assessing protective factors using the SAPROF or other strength-based assessment tools for treatment and promoting re-integration into society among forensic psychiatric patients are warranted.

### Limitations

This study has some limitations. First, while both the HCR-20 and SAPROF were mainly based on a psychiatric evaluation report recorded by a psychiatrist, a life and environmental report recorded by a probation officer, and clinical records of multi-disciplinary professionals within the first 2 weeks following hospitalization, the HCR-20 was assessed by the psychiatrist in charge so that the psychiatrists could begin seeing the patients. Second, the study design, along with the short observation period for the initial assessment (i.e., within 2 weeks of hospitalization) on the clinical records, might have negatively impacted interrater reliability, as the ICCs of coping, leisure activities, financial management, and motivation for treatment were all < 0.4. Third, the data on the occurrence of violence were limited to incidents reported in the electronic clinical records. Fourth, the sample size was insufficient to allow sub-group analyses. Therefore, we cannot determine the predictive validity of the SAPROF for men and women separately and in different diagnostic categories. Finally, the applicability of our results is limited to forensic psychiatric inpatients admitted under the MTSA in Japan. The predictive accuracy of the SAPROF among forensic outpatients, general psychiatric patients, or individuals in other forensic settings in Japan is unknown.

## Conclusion

The SAPROF Japanese version, a structured professional judgment tool focused on the protective factors against violence, is an effective tool as a significant predictor of desistance from violent behavior among Japanese forensic psychiatric inpatients. The SAPROF might allow for a more balanced assessment of future violence risk in places where the population rates of violent crime are low, such as Japan, but a validation study in a different setting is needed to confirm this.

## Abbreviations

AUC: area under the curve
HCR-20: Historical Clinical Risk Management-20
ICC: intraclass correlation coefficient
ICD-10: International Classification of Diseases, Tenth Edition
MTSA: Medical Treatment and Supervision Act
ROC: receiver operating characteristic
SAPROF: Structured Assessment of PROtective Factors
